# Recurrent myocardial infarction: Mechanisms of free-floating adaptation and autonomic derangement in networked cardiac neural control

**DOI:** 10.1371/journal.pone.0180194

**Published:** 2017-07-10

**Authors:** Guy Kember, Jeffrey L. Ardell, Kalyanam Shivkumar, J. Andrew Armour

**Affiliations:** 1 Dept. of Engineering Mathematics and Internetworking/Faculty of Engineering/Dalhousie University, Halifax, NS, Canada; 2 David Geffen School of Medicine/Cardiac Arrhythmia Center, University of California – Los Angeles (UCLA), Los Angeles, CA, United States of America; Georgia State University, UNITED STATES

## Abstract

The cardiac nervous system continuously controls cardiac function whether or not pathology is present. While myocardial infarction typically has a major and catastrophic impact, population studies have shown that longer-term risk for recurrent myocardial infarction and the related potential for sudden cardiac death depends mainly upon standard atherosclerotic variables and autonomic nervous system maladaptations. Investigative neurocardiology has demonstrated that autonomic control of cardiac function includes local circuit neurons for networked control within the peripheral nervous system. The structural and adaptive characteristics of such networked interactions define the dynamics and a new normal for cardiac control that results in the aftermath of recurrent myocardial infarction and/or unstable angina that may or may not precipitate autonomic derangement. These features are explored here via a mathematical model of cardiac regulation. A main observation is that the control environment during pathology is an extrapolation to a setting outside prior experience. Although global bounds guarantee stability, the resulting closed-loop dynamics exhibited while the network adapts during pathology are aptly described as ‘free-floating’ in order to emphasize their dependence upon details of the network structure. The totality of the results provide a mechanistic reasoning that validates the clinical practice of reducing sympathetic efferent neuronal tone while aggressively targeting autonomic derangement in the treatment of ischemic heart disease.

## Introduction

From an autonomic perspective, unstable angina and recurrent myocardial infarction (MI) overlap in a mechanistic sense and, as such, preventing their pathological adaptation during the progression of heart disease would relieve a significant health burden [1]. While population studies do not typically address the autonomic nervous system in a direct fashion, it is understood that the autonomic nervous system plays a role in the production of ventricular arrhythmias and sudden cardiac death ([2] and [3]).

**Fig 1:**
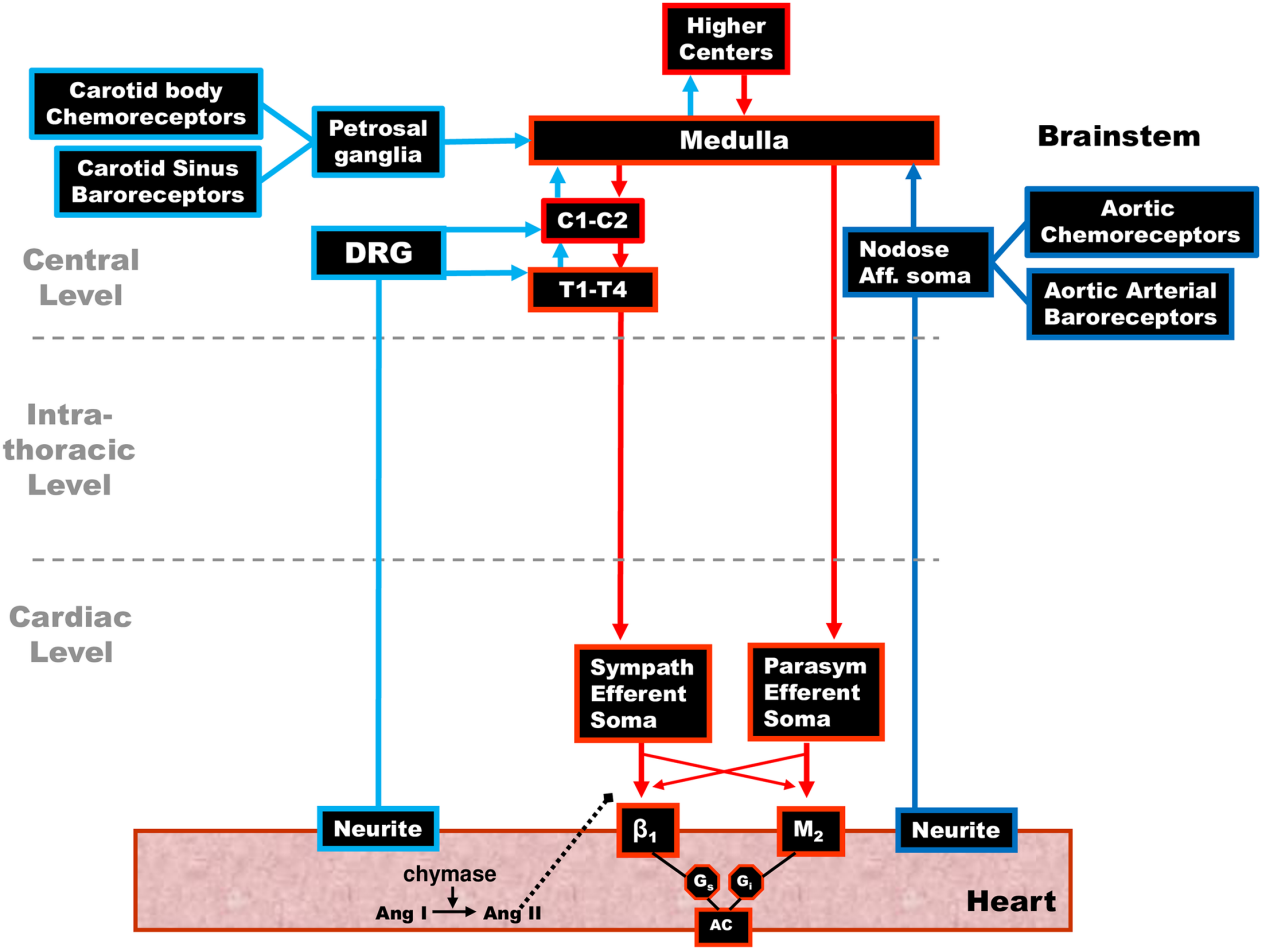
Central model of cardiac control. The classical view of the neuronal populations making up the cardiac neuronal hierarchy. In this view the cardiac neuroaxis is made up of two main neuronal groupings based on their anatomic locations: i) higher center neurons, including those in the medulla and spinal cord (C1-T4 primarily) up to the level of the insular cortex. ii) peripheral ganglia—a) neural somata in intrathoracic extracardiac ganglia (adrenergic postganglionic motor neurons) and b) those on the heart (cholinergic postganglionic motor neurons). In this model, these peripheral neuronal populations are under the control of central neuronal command.

**Central Model of Cardiac Regulation.** In the “classical” central model of cardiac regulation depicted in Fig 1 the central nervous system processes cardiac specific and more general cardiovascular/homeostatic control requirements. This central control paradigm primarily encompasses the spinal cord, medullary and higher center reflex processing and the peripheral nervous system is used as a conduit to channel centrally-computed inputs to the cardiac level. Circulating blood volume (equivalently, blood flow or cardiac output) is managed by centrally controlled resetting of the baroreflex and afferent inputs from the exercise pressor reflex ([4, 5], the latter is not shown in Fig 1). In this framework, the central nervous system and skeletal muscle feedback is used to reset baroreceptor reflexes and the peripheral nervous system acts to relay motor inputs to the cardiac level ([4, 6]). A useful summary, and questions surrounding this model are elucidated in [7].

**Fig 2:**
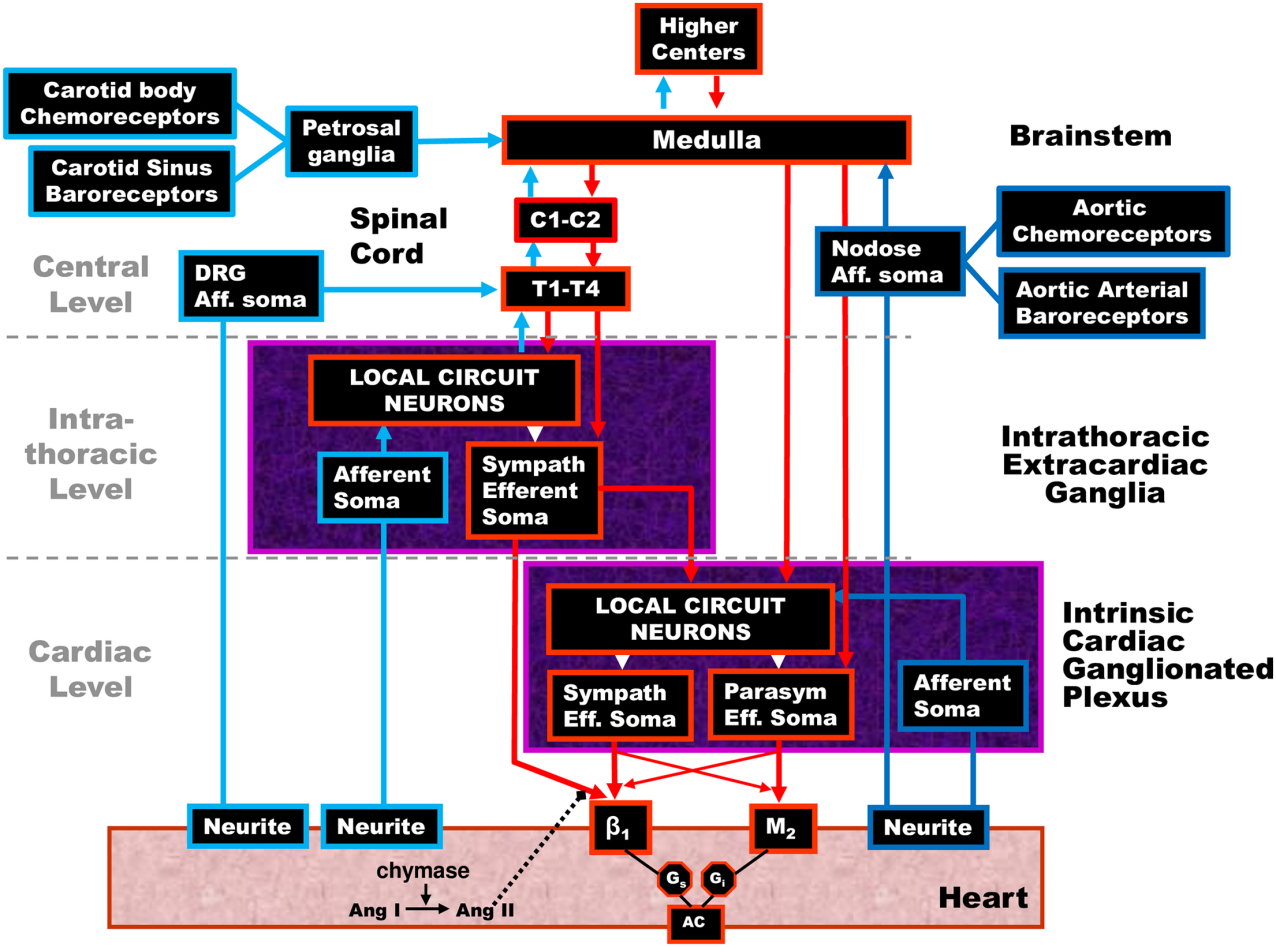
Hierarchical networked model for cardiac control. Network interactions occur within the local circuit neural (LCN) populations. These integrate activities within and between peripheral ganglia and the central nervous system subserve reflex control of the heart. The intrinsic cardiac nervous system possesses sympathetic (Sympath) and parasympathetic (Parasym) efferent post-ganglionic neurons, local circuit neurons (LCN) and afferent (Aff.) neurons. The intrathoracic extracardiac nervous system is comprised of ganglia containing afferent neurons, LCN and sympathetic efferent post-ganglionic neurons. Cardiovascular heart rate and demand inputs are conveyed centrally via dorsal root (DRG), nodose and petrosal ganglia subserving spinal cord (C-cervical, T-thoracic), brainstem and higher center reflexes for hemostatic maintenance.

**Networked Model of Cardiac Regulation.** From the perspective of investigative neurocardiology, recent animal models have shown that cardiac regulation involves a multiple-level hierarchy of cardiac control that reaches from higher brain centers to the level of the heart as depicted in Fig 2 (a review is in [8]). All features of the central model (Fig 1), are fully contained in this expanded networked model. In this networked control model the role of the peripheral nervous system is broadened to include these additional components: (i) networked cardiac control where local circuit neurons (LCNs; interneurons) serve as centers of integration; (ii) sensory neurite feedback to afferent somata that project centrally via nodose and dorsal root ganglia; and (iii) sensory neurite feedback at the cardiac and intrathoracic levels through LCN populations. The LCN populations and feedback transduced by afferent somata are the two cornerstones of intrathoracic reflexes mediated by a) the intrinsic cardiac nervous system (ICNS) and b) intra-thoracic nervous system (ITNS). Specifically, it has been shown that the ICNS has the capability to exert bidirectional integrated reflex control of cardiac electrical and mechanical function, even when functioning disconnected from all higher neural elements of the cardiac nervous system ([9] and [10]). Furthermore, intrathoracic reflex control of the heart is partially maintained following decentralization of stellate ganglia bilaterally [11]. In other words, the ICNS and ITNS are capable of a degree of autonomous function with respect to central inputs.

The ICNS itself is comprised of distributed aggregates of ganglionated plexi, each with a preferential sphere of influence ([12] and [13]). Among these ganglia, common shared inputs (afferent and efferent), inter- and intra-ganglionic interconnections mediated by LCN’s sub-serve and assure beat-by-beat coordination of regional cardiac (electrical and mechanical) indices ([11] and [12]). Both the intrinsic and intrathoracic nervous systems receive central inputs and afferent inputs that represent a complex mix of information from the heart, lungs, carotid and aortic baroreceptors, viscera, periphery and a host of other factors. Each of these processing centers interact locally and in an interdependent fashion with neurons in other levels to coordinate regional cardiac indices on a beat-to-beat basis.

**Clinical Relevance.** Given an active role for the peripheral nervous system in cardiac regulation as indicated from investigative neurocardiology, it is reasonable to anticipate that autonomic involvement in pathology will follow subtle and complex pathways ([14] and [15]). To date, it is clear that the autonomic nervous system affects and is affected by cardiovascular disease (CVD). Significant interactions between central and peripheral reflexes influence the potential for cardiac arrhythmias, [3] the risk of sudden cardiac death, and the progression into heart failure [16]. These interactions are important because the autonomic nervous system, by default, must continue to exert closed-loop control over the heart and its associated elements in the presence of pathology. Because this hierarchy is also extremely adaptable at the molecular, cellular and global levels ([2, 8], and [17]) its capacity for remodelling, while beneficial in the absence of pathology, can go awry during the evolution of pathology.

**Study Aims.** In this study, a mathematical model for open-loop networked cardiac regulation [15] is extended to closed-loop control and canonical models for recurrent MI and/or unstable angina are developed. A benchmark balance, or signature, is found that represents the relative tone of the components within this control system in the presence and absence of pathology. The concept of a benchmark balance is used here to allow comparison of the networked cardiac control status in the prelude and aftermath of recurrent pathology. It represents the relationship between central demand, sympathetic tone, and parasympathetic tone in the closed-loop networked control response to a steady demand both in the prelude and the aftermath of the recurrent pathology. As such, the response to pathology begins from an existing benchmark balance, in the prelude to pathology, that is followed by a dynamical response ending at a new benchmark balance that represents a new normal in the aftermath of the pathology. Both the final benchmark balance and the dynamics of the pathways explored by the network while moving to the new normal are examined in terms of the network structure, network cell diversity, and susceptibility to autonomic derangement.

## Materials and methods

### Mathematical model and anatomy of closed-loop control

The open-loop form of the model developed in [14, 15], and [18] is generalized here to closed-loop cardiac regulation. In addition, canonical models are constructed for recurrent MI and unstable angina pathologies. To elucidate concepts, the mathematical model is briefly described below and this is followed up with equations that generalize the open-loop form to a closed-loop control. The canonical models for recurrent MI and unstable angina appear in later sections.

Referring to Fig 2, the central model in Fig 1 is generalized to a model of cardiac regulation that includes a networked peripheral nervous system. Local circuits comprise the ITNS (intrathoracic/extracardiac nervous system) and ICNS (cardiac/intrinsic cardiac nervous system). Inputs to ganglionic LCN populations arise both centrally and from various sensory neurites associated with afferent somata that are associated with carotid and aortic baroreceptor neurons, as well as those located in the heart, lungs, viscera, periphery, etc., All of these are integrated before being passed to the efferent nervous system. Critical feedback is derived from cardiac sensory neurites associated with afferent neuronal somata located at the cardiac, intra-thoracic, and central levels.

**Fig 3:**
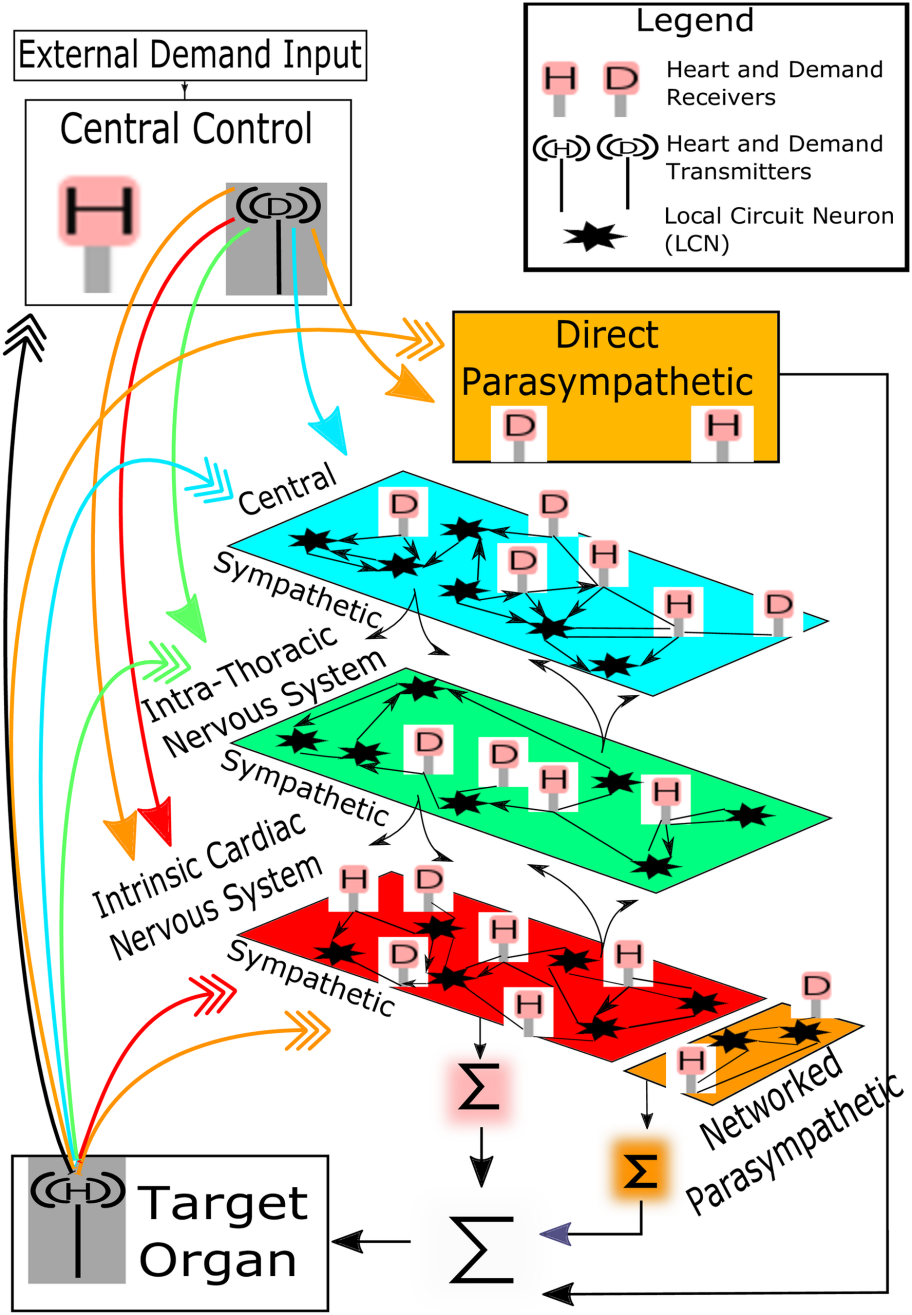
Schematic of mathematical model. Closed-loop control of cardiac output is shown as a networked 3-level hierarchy.

A schematic of the mathematical model for closed-loop, networked cardiac regulation is represented in Fig 3. The model is a canonical form of the three-level control hierarchy depicted in Fig 2. As such, the mathematical model presented here is evolved from [15] and used to develop a greater understanding of hierarchical networked control and related responses to disease pathology. The model is particularly useful in that it provides a means to study such pathologies that may prove difficult experimentally due, in part, to the current technical limitations of concurrently assessing the function of distributed neuronal populations *in situ.*

The three levels within this control hierarchy are: (i) central (blue) or ‘top’ level, (ii) ‘middle’ (green) or intrathoracic/extracardiac level, and (iii) the cardiac (red) or ‘bottom’ level. The red, green, and blue colorations refer to the sympathetic nervous system, while the remaining parasympathetic neural components, outlined below, are colored in brown.

The heart is depicted at the bottom of Fig 3. In the model, the heart is reduced to a first-order responder and a constant ejection fraction is assumed so that cardiac output is approximately proportional to heart rate [15]. Thus, within the closed-loop control shown in Fig 2, heart rate is the controlled variable. The setpoint for heart rate is varied to meet global demands for cardiac output and treated as a lumped quantity. As such, the setpoint is labelled as ‘External Demand Input’ in Fig 3 and the term ‘External’ is to emphasize that setpoint determination is lumped and external to the model. While the setpoint used is found externally to the model, blood flow demands required to meet the external setpoint are modelled and these demands (equations appear below) are computed within ‘Central Control’ in the figure.

Inputs to the network are received by neurons sensitive to global blood flow demand and by those sensitive to cardiac status taken as heart rate in the model. The study of anatomy and function [9] indicates an arrangement where both types of neurons are spread throughout the network, with proportionally more blood flow demand neurons located centrally and a greater percentage of heart rate neurons to be found at the cardiac level ([14] and [15]). This centric arrangement reflects a coupling between anatomy and function with a cardiac-centric bottom level and a centrally-centric top level ([4] and [19]) while the middle portion is assumed to occupy an intermediate and overlapping role.

Although the format and source of blood flow demand inputs and information surrounding cardiac status is level-centric, a simplification is made within the mathematical model where a single form of global demand and cardiac status (taken as heart rate in the model) is respectively transmitted from central control and the target organ. This is shown in Fig 3 where blood flow demand and heart rate is transduced. Likewise, neurons within the network that receive demand inputs are modelled as ‘D’ receivers and these are relatively common at the central level as shown in Fig 3. Similarly, those neurons receiving heart rate are ‘H’ receivers and there is a larger population of these at the cardiac level (see Fig 3). Feedforward from the central control blood flow demand transducer is depicted as a destination colored arrow with a solid head while feedback of heart rate is shown in the same fashion with multiple arrowheads.

Neurons within the network that do not receive either blood flow demand or heart rate information are networked to form local circuits (stars in Fig 3). Within this population the adrenergic sympathetic neural population is networked (shown as the red, green, and blue colored levels of Fig 3). The cholinergic parasympathetic motor neurons are in two parts: (i) a ‘direct’ vagal component termed ‘direct parasympathetic’ (brown colored, direct parasympathetic component in Fig 3) that is not networked but runs from the central control structure directly to the target organ (bottom of Fig 3), and (ii) an ‘indirect’ networked component (brown colored networked parasympathetic in Fig 3) at the cardiac level (adjacent to the sympathetic ‘red’ colored portion in the figure). This networked parasympathetic component has been identified as existing within the intrinsic cardiac nervous system [8] and has been previously explored [15]. Efferent motor control inputs to the target organ are shown as a collection of *Σ* terms in Fig 3. These are modelled as a single lumped-parameter, with efferent input equal to a linear combination of: (i) averaged sympathetic and parasympathetic efferent postganglionic neuronal activities represented at the networked cardiac level of the network, and (ii) direct parasympathetic efferent neuronal activity.

All neurons, including heart rate and blood flow demand neurons, are influenced by neighbouring neuronal activity and network adaptibility is modelled through homeostatic and Hebbian plasticity [18].

The closed-loop component of the model, that was not previously explored in [15], is assumed to follow a parsimonious proportional-integral (PI) control scheme. Employing the same notation that was used in [15] the central control blood flow demand *D*^(*n*)^ at time interval *t*_*n*_ is applied over times *t*_*n*_ ≤ *t* ≤ *t*_*n*+1_ and *t*_*n*+1_ − *t*_*n*_ = Δ*t* is the constant simulation time interval. From the standard PI control rule, the blood flow demand beginning at time *t*_*n*_ is

$$\begin{array}{c} D^{\left(n\right)}=K^{\left(PI\right)}(H^{(n)*}-H^{\left(n-L\right)}+\frac{1}{T_{i}^{\left(PI\right)}}\sum_{j=0}^{n}(H^{(j)*}-H^{\left(j-L\right)})\Delta t) \end{array}$$ (1)

*H*^(*n*)^* is the heart rate setpoint over time interval *t*_*n*_, *H*^(*n*−*L*)^ is the heart rate delayed by a time *t*_*L*_ = *L*Δ*t* and the PI-control parameters are a constant control gain *K*^(*PI*)^ and a constant ‘reset time’ $T_{i}^{\left(PI\right)}$. The setpoint *H*^(*n*)^* is determined externally to central control within ‘External Demand Input’ (Fig 3). The blood flow demand *D*^(*n*)^ received by the network is chosen within ‘Central Control’ (Fig 3) such that the difference between the setpoint and delayed heart rate, *H*^(*n*)^* − *H*^(*n*−*L*)^, is minimized. The gain *K*^(*PI*)^ is maintained at a constant level for each neuron. Although the controller gain could be either time-dependent or state-dependent, such dependence is not found to be necessary within the study considered here. Such a generalization may be useful in future studies of neural networks populated by neurons that exhibit an identity [20].

### Simulation design

The closed-loop control system described above is subjected to either: (i) unstable angina or (ii) myocardial infarction while still exerting control over heart rate. Within each simulation there are three *episodes* *E*_*j*_, *j* = 1, 2, 3 of either unstable angina or infarction. Each episode contains three contiguous sub-events *I*_*j*_, *j* = 1, 2, 3, *R*_*j*_, *j* = 1, 2, 3 and *D*_*j*_, *j* = 1, 2, 3. The *j*^*th*^ episode is *E*_*j*_ = (*I*_*j*_, *R*_*j*_, *D*_*j*_), *j* = 1, 2, 3 and *E*_1_ occurs first, *E*_2_ is second and *E*_3_ is last. After the last episode there is an aftermath period, beginning at the vertical dashed line, where the population-wide sensitivities of neurons transducing blood flow demand and heart-rate are re-initialized to the same levels identified on average before *E*_1_ and the system is allowed to seek a new normal. The form of the sub-events given below is intended as a highly simplified representation containing elements that are generic to an infarction or angina event. While the clinical reality is much more complex, the aspects considered here are used to provide insight into the nature of interactions between networked cardiac control and recurring pathologies.

1. Sub-event 1: Infarction or angina event occurring at *I*_*j*_, *j* = 1, 2, 3
  Each sub-event occurring at times *I*_*j*_, *j* = 1, 2, 3 is associated with increased drive at the cardiac level of the network [14] beginning at each of three times labelled as *I*_1_, *I*_2_ and *I*_3_ and respectively ending at the beginning of sub-event 2.
2. Sub-event 2: Recovery beginning at *R*_*j*_, *j* = 1, 2, 3
  Following the infarction or angina there is a recovery beginning at times labelled *R*_1_, *R*_2_ and *R*_3_ and the network is allowed to move to a new steady state.
3. Sub-event 3: External Demand occurring at times *D*_*j*_, *j* = 1, 2, 3
  After the recovery phase a mild increase in central control drive, to increase heart rate, is applied at times *D*_1_, *D*_2_ and *D*_3_.

**Fig 4:**
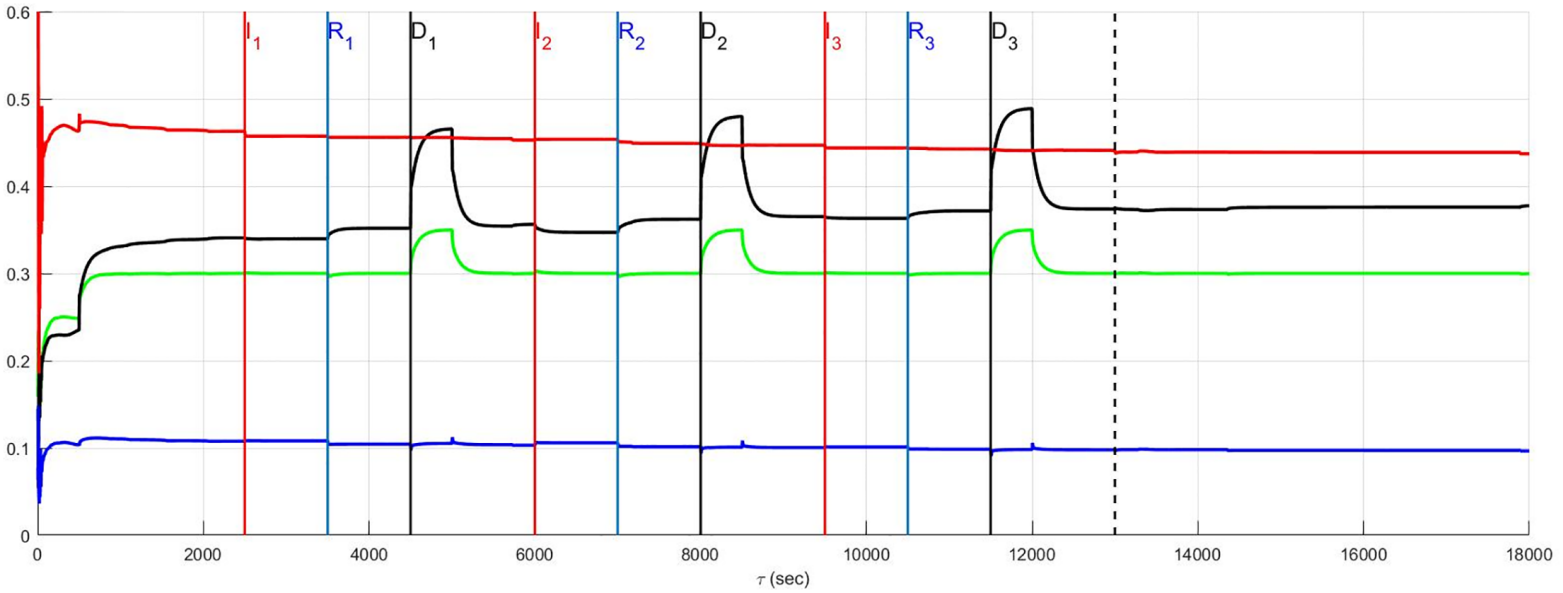
In Fig 4 through Fig 14 inclusive the simulation results are depicted as solid lines for four variables: (i) efferent sympathetic activity (red), (ii) heart rate (green), (iii) central drive (black), and (iv) parasympathetic efferent (blue). The simulation timeline definitions described here are further explained in Simulation Design. Each of the red vertical lines *E*_*j*_, *j* = 1, 2, 3 indicates the beginning of the *j*^*th*^ episode. Three sub-events within each episode are the red, blue, and black vertical lines that respectively correspond to the onset of infarction (or unstable angina), recovery, and demand. The last vertical dashed line indicates the beginning of the aftermath of the recurrent pathology. In this figure, a stratified network with low neural diversity is shown. Local circuit neurons are affected and remain alive. Neurons that transduce heart rate and blood flow demand are affected and remain alive. There is no autonomic derangement.

The simulation of the recurrent pathology is designed such that the timeline *between* each episode may be arbitrarily expanded without changing the results since a steady-state of the system is approximately achieved after each episode has ended. In Fig 4 through Fig 14 inclusive, efferent sympathetic activity (red), heart rate (green), central drive (black), and parasympathetic efferent (blue) are depicted.

### Simulation timeline

The detailed simulation time line is as follows

1. Initialization: The system is initialized from 0 ≤ *t* < 500 seconds with the heart-rate setpoint *H*^(*n*)^* = 0.25.
2. Steady-State: The system is allowed to approximate a steady-state while controlling the heart rate to a constant level *H*^(*n*)^* = 0.3 during 500 ≤ *t* < 2500 seconds and this setpoint is used throughout the simulations except for times when a mild drive is applied for 500 seconds starting at times *D*_*j*_, *j* = 1, 2, 3 when *H*^(*n*)^* = 0.35.
3. Infarction or angina: Three episodes of pathology have the timelines depicted below
  - Episode 1: *I*_1_ over 2500 ≤ *t* < 3500 seconds, *R*_1_ 3500 ≤ *t* < 4500 seconds, *D*_1_ 4500 ≤ *t* < 5000 seconds, and move to steady-state during 5000 ≤ *t* < 6000 seconds
  - Episode 2: *I*_2_ over 6000 ≤ *t* < 7000 seconds, *R*_2_ 7000 ≤ *t* < 8000 seconds, *D*_2_ 8000 ≤ *t* < 8500 seconds, and move to steady-state during 8500 ≤ *t* < 9500 seconds
  - Episode 3: *I*_3_ over 9500 ≤ *t* < 10500 seconds, *R*_3_ 10500 ≤ *t* < 11500 seconds, *D*_3_ 11500 ≤ *t* < 12000 seconds, and move to steady-state during 12000 ≤ *t* < 13000 seconds
  - Aftermath and a New Normal State: The blood flow demand and heart rate neural population sensitivities are re-initialized to the same statistical properties that existed before the recurrent pathology. The system is allowed to establish a new normal following re-initialization in the aftermath of the three episodes. This occurs from 13000 < *t* ≤ 18000 seconds while controlling heart rate to the original setpoint *H*^(*n*)^* = 0.3 without any pathological influence.

### Modelling context

During all simulations this hierarchical system remains in closed-loop control such that central control attempts to maintain ‘heart rate’ at a setpoint. Both recurrent infarction and unstable angina are considered as a function of four variables. Two of the variables deal with network structure and these include the global preferential synaptic connectedness within the network and the degree of network neural diversity. The remaining two variables deal with neural pathology in neurons that transduce heart rate and blood flow demand, along with the LCN neuronal populations.

The two variables dealing with network structure are

1. Network Connectedness.
  There are two cases considered
  - Stratified.
    In a ‘stratified’ network neurons are preferentially connected to peer neurons. Stratified networks have a 10:1 ratio of peer-to-peer:adjacent layer connections throughout the hierarchy.
  - Top-Down.
    A ‘top-down’ network structure has neurons typically connected to adjacent layers. This network structure is in direct contrast with the stratified form and is assumed to have a 1:1 ratio of peer-to-peer:adjacent layer connections. Note that a choice of 1:10 ratio of peer-to-peer:adjacent layer connections, to complement the stratified network defined above, would reduce the top-down network structure to effectively another version of a stratified network and therefore this case is not considered herein.
  In both the stratified and top-down networks there is no more than one adjacent layer connection allowed from lower to higher levels such that information primarily flows downward from the top to the bottom of the network.
2. Neural Diversity.
  The measure of neural diversity employed in this model is directly proportional to the relative number of heart-rate and blood flow demand neurons, as compared with local-circuit neurons. Two limiting cases are considered
  - Low Diversity.
    At low neural diversity, each layer has 10% neurons that transduce heart rate and blood flow demand with the demand:heart-rate neurons 5:1 at the top or central level, 1:1 at the middle or intrathoracic level and 1:5 at the cardiac level. The remaining 1800 neurons (there are 600 neurons/layer) within each of the three levels are local circuit neurons.
  - High Diversity. At high diversity the number of neurons that transduce heart rate and blood flow demand relative to local circuit neurons is increased to approximately 50% at all levels with the same 5:1, 1:1, 1:5 ratio for demand:heart-rate neuron ratios used in the low-diversity case.

The second pair of variables relate to pathological circumstances that define neural pathology as either: (i) neurons are affected by infarction or are affected by ischemia, and (ii) autonomic derangement of heart rate and blood flow demand neuron sensitivity occurs in the absence of neuron death [14].

1. Neurons affected by ischemia or infarction.
  During each episode beginning at times *t* = *I*_*j*_, *j* = 1, 2, 3 affected neurons may be chosen from both those transducing heart rate and blood flow demands, and local circuit neuronal populations. For both populations, approximately 15% are chosen from the cardiac level, 4% from the intrathoracic and 2.5% from the central level.
  In the case of myocardial infarction the affected neurons are assumed to die, while during periods of unstable angina neurons remain alive. When the affected neurons remain alive their activity is *outside* central control or the influence of network neighbours; their activity remains, as evident just before the beginning of each episode so that these neurons continue to influence the network behaviour.
2. Autonomic Derangement.
  Changes in sensitivity of neurons receiving blood flow demand and heart-rate feedback have the capacity to trigger harmful changes in the benchmark balance between central drive and sympathetic tone. The endpoint of these changes and the dynamics of the pathways are also less predictable than the effects of recurrent pathology considered so far. Autonomic derangement is present when heart-rate neurons experience an approximately 25% inappropriate decrease in sensitivity to feedback and blood flow demand neurons a 25% increase in sensitivity to central demands [14]. The derangement commences with each new ischemic episode at *I*_*j*_, *j* = 1, 2, 3 and ends at the beginning of the associated recovery phase *R*_*j*_, *j* = 1, 2, 3. Such derangements accumulate between episodes such that in the aftermath phase the population of heart-rate and blood flow demand neurons are re-initialized to their average status of the population before any derangement occurred.

## Results and discussion

### Benchmark balance: Unstable angina without autonomic derangement

The benchmark balance is an operational reference level that exists in the absence of pathology. It is characterized by the functional balance among neurons that transduce heart rate and blood flow demand, local circuit neuronal populations and between various levels of the network that control cardiac efferent neuronal outputs. This balance is important because it is a baseline from which the network evolves during each attended state. The benchmark used here is taken to be the neural network balance among the sympathetic efferent, direct parasympathetic efferent, and central neuronal command drive *D*^(*n*)^. The network balance is investigated here as a function of the network structural characteristics, stratified or top-down, and neural diversity of the populations involved.

### Stratified network and neural diversity

**Fig 5:**
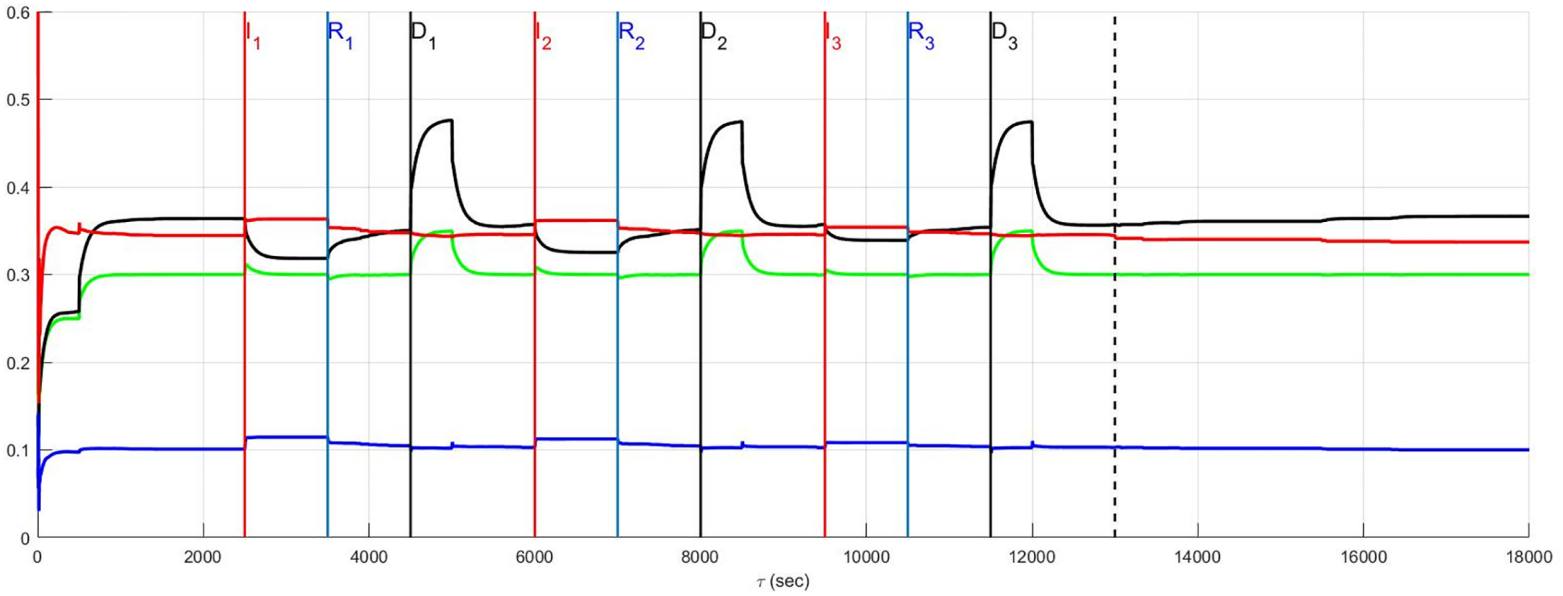
A stratified network with high neural diversity is shown. Local circuit neurons are affected and remain alive. Neurons that transduce heart rate and blood flow demand are affected and living. There is no autonomic derangement.

The effect of neural population diversity is shown in Figs 4 and 5 for low and high neural diversity respectively within a stratified neural network. The stress employed is representative of three repetitive, transient ischemic events. The benchmark balance, before the onset of angina, can be seen for this stratified network after approximately 500 seconds have elapsed, for times 500 ≤ *t* < 2500 seconds when the sympathetic and parasympathetic motor tones are, respectively, approximately 0.5 and 0.1 and the central control drive is approximately 0.35. The network considered in Fig 4 has low neural diversity such that comparisons over the same times may be presented as in Fig 5 where a stratified network is depicted with high neural diversity. At high neural diversity, the central control drive is somewhat higher but the sympathetic tone is reduced by 30% to approximately 0.35. Hence, there is a heightened central drive coupled with lowered sympathetic tone compared to that seen in the low-diversity stratified network. This high-diversity balance represents a more resilient network in the sense that it is better positioned to provide increased cardiac output when needed and without excessive sympatho-excitation.

At and beyond *t* = 2500 seconds, in Figs 4 and 5, each episode *E*_*j*_, *j* = 1, 2, 3 begins at the labels representing the first, second and third event (*I*_1_, *I*_2_ and *I*_3_) respectively, with neurons transducing heart rate and demand as well as LCN populations being affected. All of the affected neurons survive and there is no autonomic derangement throughout the simulation.

The most important effect of the ensuing ischemia is that of the benchmark balance between central control drive, sympathetic efferent along with direct parasympathetic efferent neuronal outputs that persist. In both the high and low diversity networks the benchmark balance persists throughout the simulations such that the high diversity network consistently has lower sympathetic tone. In fact, in both of these simulations there is a increasing improvement in the network response to the angina, i.e. there is a reduction in the sympathetic efferent neuronal activation and a concurrent increase in central control drive after each episode. This improvement is due to network synaptic plasticity [15] that serves to reduce sympatho-excitation.

A result such as this does not imply that unstable angina is of any clinical benefit. Rather, the main observation is that it is possible for a resilient neural network to find a way, in the absence of other confounding influences, to maintain function when faced with a pathology such as unstable angina. Although confounding influences that are part of cardiovascular disease cannot be simply removed, the results do imply that this neural network can be expected to maintain its internal balance under such circumstances. Note that this result is also referenced in the summary Fig 15 where the main results surrounding the relationship between the benchmark balance in the prelude and aftermath are highlighted.

### Top-down network and neural diversity

**Fig 6:**
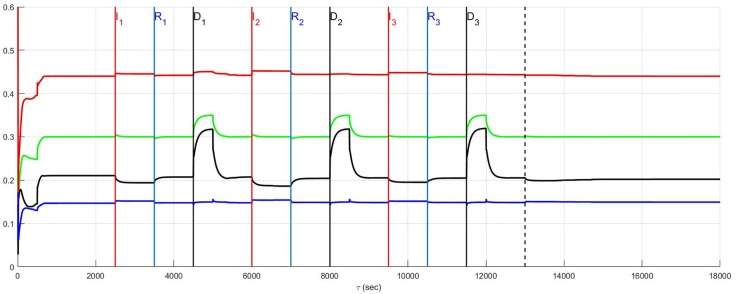
A top-down network with low neural diversity. Local circuit neurons are affected and survive. Neurons that transduce heart rate and blood flow demand are affected and also survive. There is no autonomic derangement.

**Fig 7:**
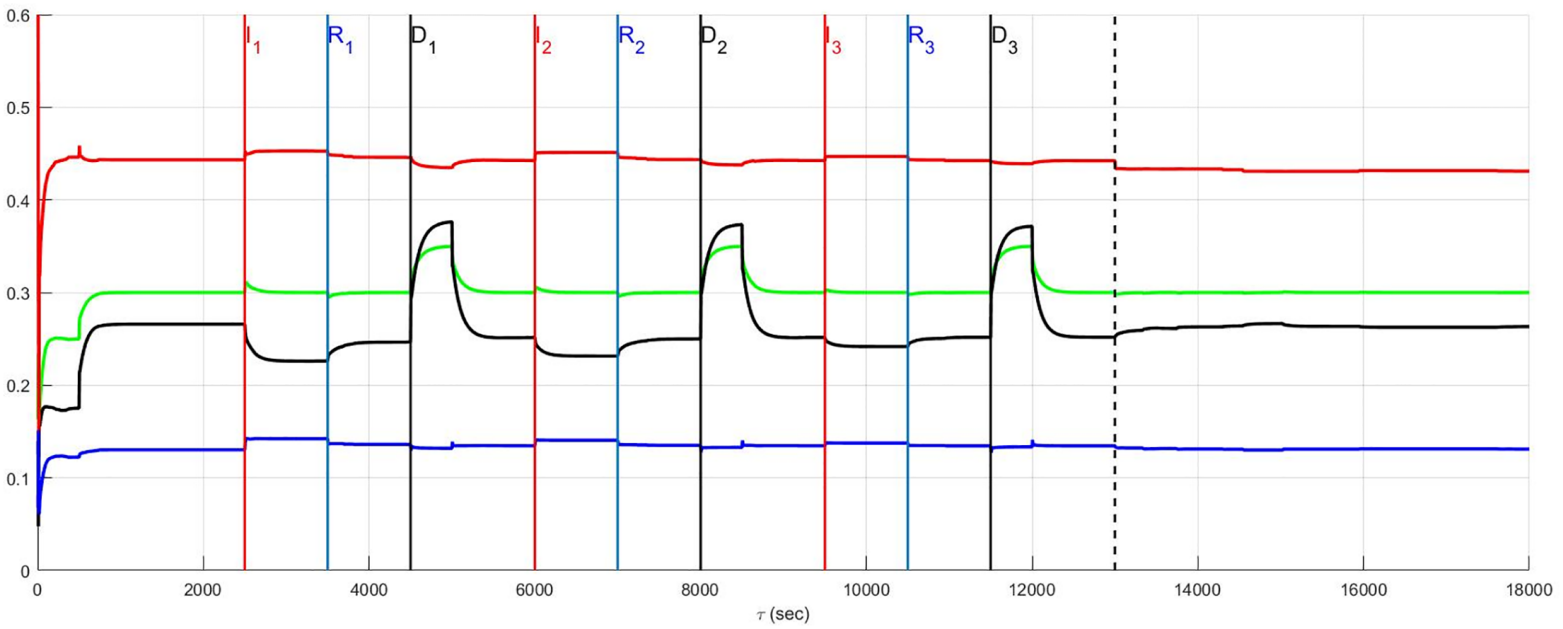
A top-down network with high neural diversity. Local circuit neurons are affected and remain alive. Neurons that transduce heart rate and blood flow demand are affected and survive. There is no autonomic derangement.

The stratified network simulations depicted in Figs 4 and 5 are repeated for a top-down network configuration in Figs 6 and 7. Once again the stressor is repeated myocardial ischemic events. In these figures the benchmark balance achieved over 500 ≤ *t* < 2500 seconds is demonstrated to be approximately the same. However, unlike stratified networks the top-down network is rigid and shows little sensitivity to low and high neural population diversity. Information passes from the central to cardiac levels with relatively little influence from peer neighbours compared to what was identified in stratified networks (c.f. immediately above) such that the effect of neural diversity is ‘washed out’. Neural diversity has been found ([20, 21], and [22]) to be an important contributor to robustness in biological networks. The primary observation with respect to the model is that peer-to-peer processing of information at each level within the stratified hierarchy increases the influence of neural diversity within networked control.

As a final note, the stratified network with high diversity (Fig 5) has a consistently lower sympatho-excitation compared with the stratified low diversity and both of the top-down neural network structures. During the episodes *I*_*j*_, *j* = 1, 2, 3 there is a small, relative sympathetic efferent elevation in the stratified, high diversity structure due to the increased sensitivity of this neural network. This elevation does not translate to a persistently higher new normal in the aftermath period extending beyond *t* = 13000 seconds but moves to new normal with a lower efferent neuronal excitation.

### Benchmark balance: Unstable angina with autonomic derangement

In the previous section (Fig 5), the unstable angina is considered without any accompanying autonomic derangement. As was discussed above, there is a general improvement in the response in the aftermath of the pathology with the emergence of a new normal that represents an improved balance among neuronal populations: this new normal elicits a lowered sympathetic efferent neuronal tone coupled to a higher central control drive. As depicted herein, any previous improvement is erased by the presence of autonomic derangement which exerts a major pathological effect on global network function.

**Fig 8:**
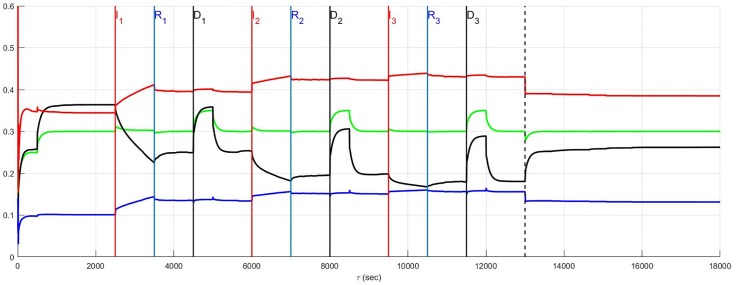
A stratified network with high neural diversity. Local circuit neurons are affected and remain alive. Neurons that transduce heart rate and blood flow demand are affected and also remain alive. There is autonomic derangement.

Unstable angina in the absence of autonomic derangement (Fig 5) is considered now in Fig 8 with autonomic derangement beginning at *t* = *I*_*j*_, *j* = 1, 2, 3 and ending at *t* = *R*_*j*_, *j* = 1, 2, 3. The derangement has a pathological and accumulating influence on neural control that increases with the passing of each episode. Both the sympathetic efferent and direct parasympathetic efferent neuronal tone rise in the aftermath period such that a new normal emerges with a persistent increased tone for both of these coupled populations associated with a lowered central neuronal command drive.

The new state that emerges after such autonomic derangement is in contrast to that observed in the absence of any neuronal derangement, such as that depicted in Fig 5. This comparison is further highlighted in the summary Fig 15. During this simulation, the control performance compares well with that seen in the absence of any derangement making heart rate a poor measure of the presence of any pathology from a neuronal perspective. Although not shown, similar results to those in Fig 8 apply to top-down networks.

**Fig 9:**
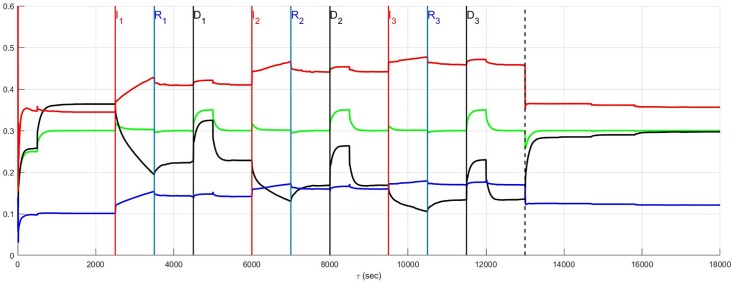
A stratified network with high neural diversity. Local circuit neurons are unaffected. Neurons that transduce heart rate and blood flow demand are affected and survive. There is autonomic derangement.

In the simulation presented in Fig 8 with autonomic derangement, cardiac neurons transducing heart rate and blood flow demand as well as LCN neurons were affected by the ischemic event. These results are followed up in Fig 9 for states in which LCN neurons are unaffected such that recurring angina does not penetrate the LCN populations. A natural assumption is that the removal of any effect on the local circuit neuronal population may be beneficial; however, this is not entirely the case in this model. When this population is unaffected (c.f., Fig 9), both sympathetic and parasympathetic efferent neuronal tone increases while central control drive is lowered *during* the three ischemic episodes (compared to that seen in Fig 8). This is due to a greater population of local circuit neurons becoming susceptible to the influence of those cardiac neurons that transduce heart rate and blood flow demand undergoing autonomic derangement. However, in the aftermath the situation is reversed such that when the local circuit neurons are unaffected (Fig 9) a reduction in sympathetic tone occurs relative to the state in which the local circuit neurons are affected (Fig 8).

From a clinical perspective, the case in Fig 8 where neurons that transduce heart rate and blood flow demand and LCN neurons are affected is likely to be closer to reality with respect to the aftermath of pathology and the initiation of, and persistent sympatho-excitation. However, an approximately 10% increase in sympatho-excitation during autonomic derangement occurs when local circuits are unaffected (Fig 9) implying that the availability of more local circuits permits corrupt feedback to ‘push’ ever deeper into the network. As a result, network function worsens the course of recovery following each pathological insult. On the other hand, referring to the summary Fig 15, the results in Fig 8 show greater sympathetic tone in the aftermath than that found in Fig 9. From a clinical perspective this implies that the form of the new normal that emerges in the aftermath of the recurrent pathology, may not be predictable from knowledge of the pathway joining the prelude and the aftermath.

### Recurrent infarction and neuron death

When recurrent infarction causes the death of intrinsic cardiac neurons perfused by the diseased coronary artery, the death of neurons even without autonomic derangement still affects network sensitivity not only to change in cardiac status but also neural diversity.

Previously (c.f., Fig 5), a progressive improvement was observed as the simulation proceeded. This occurred in the absence of any neuron death as was observed in the case for low and high diversity networks.

**Fig 10:**
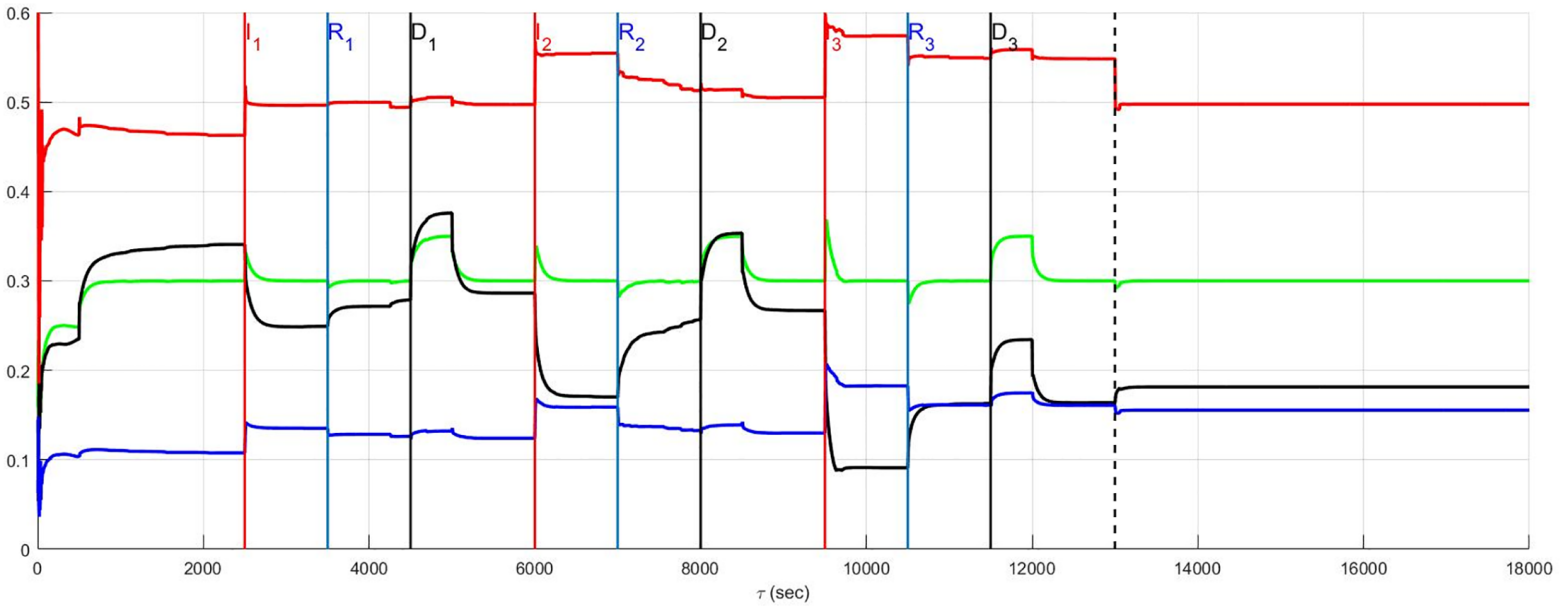
A stratified network with low neural diversity. During infarction, both local circuit neurons and neurons that transduce heart rate and blood flow demand die. There is no autonomic derangement.

**Fig 11:**
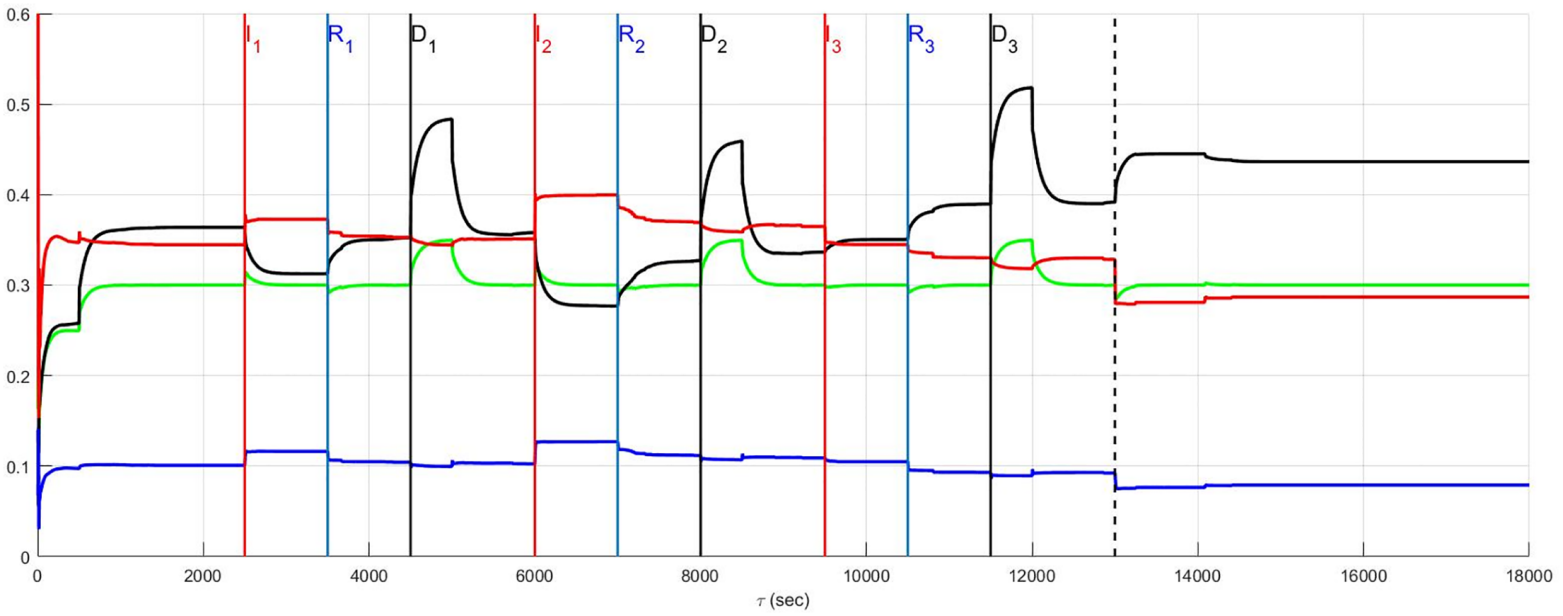
A stratified network with high neural diversity. During infarction, both local circuit neurons and neurons that transduce heart rate and blood flow demand die. There is no autonomic derangement.

The same results are reconsidered in which affected neurons, including neurons transducing heart rate and blood flow demand as well as LCN neurons (interneurons) are assumed to die. The effect of the death of these two populations is presented for low and high diversity networks respectively in Figs 10 and 11. For low diversity networks, there is a general increase in sympatho-excitation in the new normal seen in the aftermath period that contrasts with the marked decrease in the high diversity network. For low diversity networks, there are few neurons that transduce heart rate and blood flow demand and therefore the level of reduction in afferent feedback caused by heart rate and demand neuron death triggers a reduced central control drive that is compensated by an increase in sympathetic efferent neuronal tone. On the other hand, the rebalancing in high diversity networks follows a fairly complicated pathway with the aftermath period ultimately resulting in reduced sympatho-excitation.

The prelude and aftermath results in Fig 11 are also reported in Fig 15 for comparison with those previously found for high-diversity networks. The summary figure indicates that in the absence of autonomic derangement the high diversity new normal is relatively unchanged. This is despite strong differences in the pathway between the prelude and the aftermath in Figs 5 and 11.

**Fig 12:**
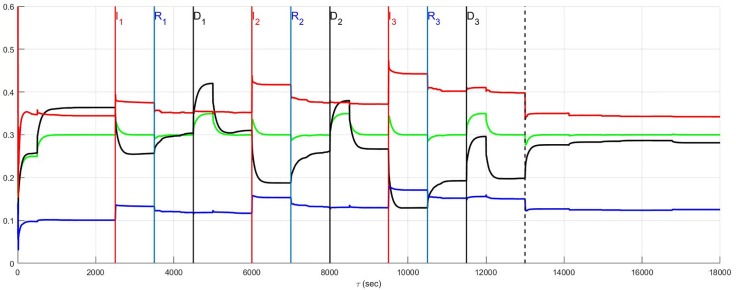
A stratified network with high neural diversity. Local circuit neurons are unaffected. During infarction, neurons that transduce heart rate and blood flow demand die. There is no autonomic derangement.

The root cause of the improvement in the aftermath in Fig 11 is unclear and a basis for understanding these results is found by reconsidering the same case with local circuit neurons unaffected (Fig 12). A comparison of Fig 11 where the local circuit neurons die during infarction and Fig 12 where these neurons are unaffected, indicates that it is the LCN death that is central to reducing sympatho-excitation in the aftermath of serial infarctions. Specifically, the death of local circuit neurons in Fig 11 leads to a compensatory increase in central control drive involving the entire hierarchy as the simulation proceeds. This increase is coupled with a reduction in sympathetic efferent tone at the cardiac level. Hence, while higher diversity of networks may minimize sympatho-excitation in the aftermath of infarctions (Fig 11), the network response remains vulnerable during the pathology to its exposure to factors depending on altered local circuit neuron function (Fig 12). The above observation implies that constructing rules-of-thumb for networked responses to recurrent pathology is unreasonable. This is primarily because free-floating adaptation of the control hierarchy depends in an unknown way on multiple factors that are, at best, difficult to measure.

### Recurrent infarction and autonomic derangement

**Fig 13:**
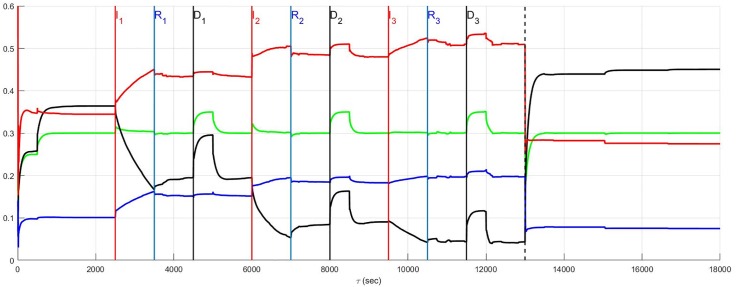
A stratified network with high neural diversity. During infarction, affected local circuit neurons and neurons that transduce heart rate and blood flow demand die. There is autonomic derangement.

Recurrent myocardial infarction with autonomic derangement [14] of a stratified high neural diversity network is depicted in Fig 13. In such a scenario, affected heart rate and blood flow demand neurons, along with LCN neurons, are assumed to die. Comparison with Fig 11 where the autonomic derangement does not occur, shows that the heart rate and demand neuron derangement leads to a large sympatho-excitation along with central control drive withdrawal.

**Fig 14:**
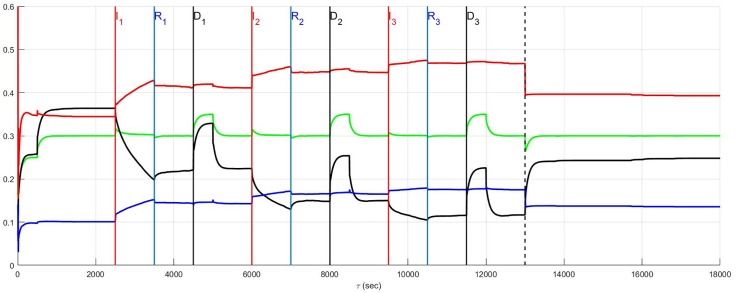
A stratified network with high neural diversity. During infarction, local circuit neurons survive while neurons that transduce heart rate and blood flow demand die. There is autonomic derangement.

**Fig 15:**
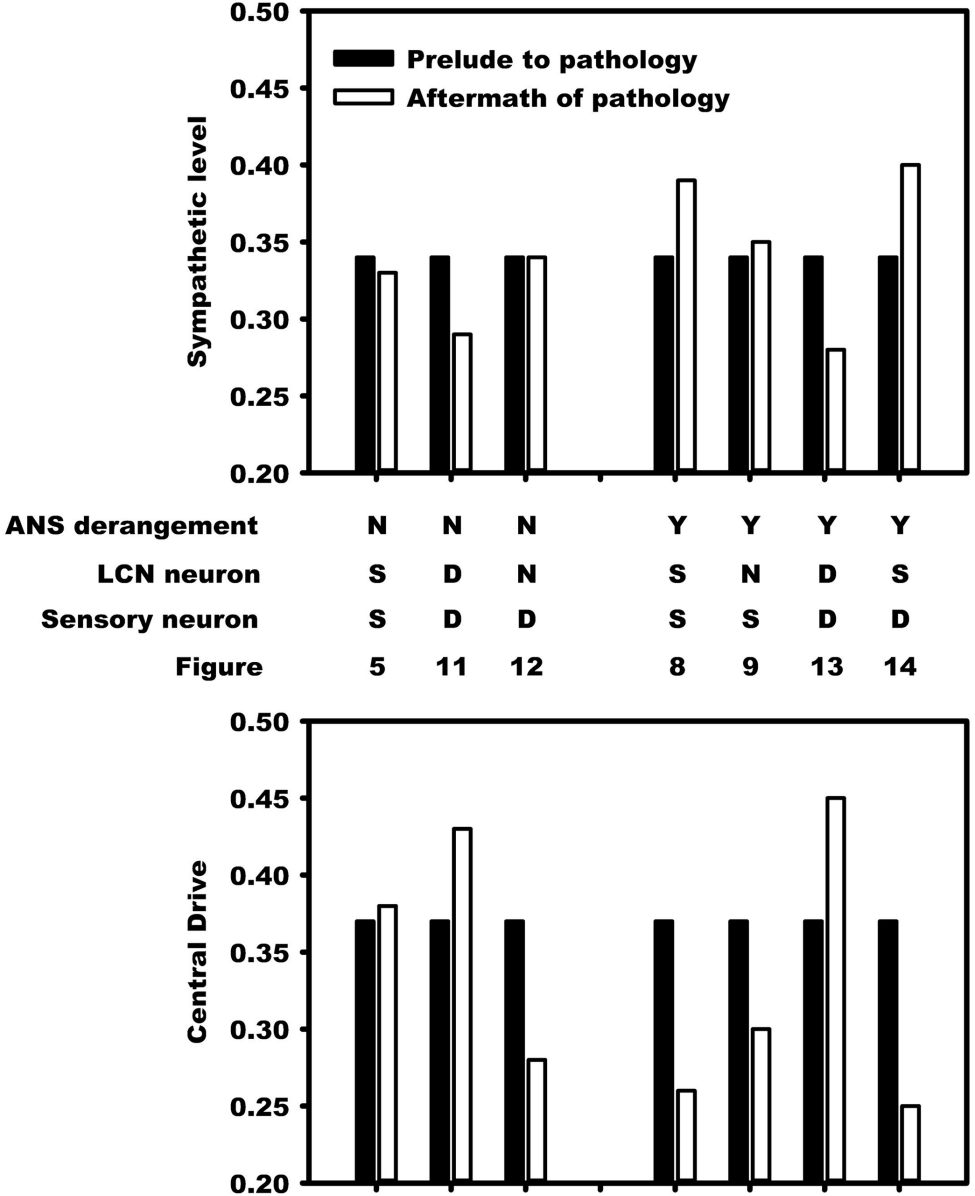
Changes that occur in this control system in response to ischemic/infarct stress predicate the degree of hyperactivity that cardiac sympathetic efferent neurons undergo. The transition of the benchmark balance from before (black bars) to the onset of recurrent MI or unstable angina and in the aftermath period of a new normal (white bars) is depicted for stratified networks with high neural diversity. The top half of the figure illustrates this transition for the sympathetic tone and the bottom half for the central drive tone. The first three pairs of bars in the top and bottom halves, starting from the left, represent the benchmark balance without autonomic derangement (’ANS derangement’ labelled ‘N’) while the remaining four pairs of bars refer to the system with autonomic derangement (’Y’). Details of how LCN neurons (’LCN Neurons’) and neurons that transduce heart rate and demand neurons are affected (’Sensory Neurons’) appears in the second and third rows. As a result of the pathology, these neurons are considered to die (’D’), become stressed (’S’), or are unaffected (’N’). In the presence of autonomic derangement (last four pairs of bars) the final state of the system becomes sensitive the extent to which local circuit neurons (’LCN neuron’) versus those transducing heart rate and blood flow demand are influenced by pathology. The vertical scales on the left represent relative neuronal activity states.

The complexity underlying the observations surrounding autonomic derangement, in this case, may be appreciated by again considering local circuit neuronal involvement. For the sake of comparison with the results depicted in Fig 13, the situation in which this population is involved in the ischemic process but now survive (c.f. Fig 14) is considered. Ischemic local circuit neurons that remain alive lead to marked increase in sympathetic efferent neuronal tone in the aftermath of the recurrent infarction in Figs 14 and 15. Yet, this result is also suprisingly coupled to a noticeable decrease in the sympatho-excitation during each pathological episode relative to that identified when local circuit neurons die (Fig 13). This complex situation is analogous to that seen in Figs 8 and 9 where the degree to which local circuit neurons are unaffected vs. affected respectively has a global influence on what occurs during and in the aftermath of the recurrent infarcts.

### Benchmark balance: Prelude and aftermath of pathology

The structural and adaptive characteristics of this networked control hierarchy are important to determine the shift towards a new normal that emerges after exposure to recurrent myocardial infarction or unstable angina. The benchmark balance, considered above relative to the dynamics for stratified networks with high neural diversity within Figs 4–14, is considered again in terms of its shift between the prelude to pathology (*t* ≤ 2500) to that seen in the aftermath of pathology (after the vertical dashed line).

The benchmark balance is summarized in Fig 15 in terms of two variables efferent sympathetic tone (top panel) and central drive (bottom panel). The dependence of these two variables on simulation details is in the four rows between the upper and lower panels. The first row indicates the presence (’Y’) or absence (’N’) of autonomic derangement (’ANS derangement’). The second and third rows respectively state whether local circuit neuron and sensory neurons transducing heart rate and blood flow demand experienced death (’D’), neural stress (’S’), and a lack of stress (’N’).

The results pre- to post-pathology are summarized in Fig 15. The change in the benchmark balance between the prelude to pathology (black bar) and the aftermath of recurrent MI and unstable angina (white bar) is shown as adjacent bars. In the absence of autonomic derangement (left three pairs of bars) the observed shifts in the benchmark balance due to neural death (’D’) are mainly accommodated by changes in central drive. In the absence of both neural death and autonomic derangement, the system returns to a state in the aftermath of pathology that approximates the state that existed before the occurrence of pathology.

However, in the presence of autonomic derangement the relationship between central drive and the sympathetic level becomes highly sensitive to the extent that neural stresses (’S’) and neural death (’D’) impact the LCN and/or neurons transducing heart rate and blood flow demand. For example, when neurons transducing heart rate and blood flow demand are affected and die (last two pairs of bars) there is a complementary system response depending upon whether local circuit neurons are either stressed (’S’) or die (’D’) as a result of the pathology. That case where LCNs are stressed, sensory neurons die, and ANS control is disrupted represents the worst case “sympatho-excitation” and most closely reflects the clinical conditions that lead to adverse outcomes ([3] and [8]).

### Model limitations

The model described here has been used to examine the dependence of the dynamics of networked cardiac control on network variables in response to recurrent pathology. Model parameters used to describe neural plasticity remain the same as those previously used to describe experimentally derived data [15] while the remaining parameters are specific to this study and empirically derived. It should also be noted that the results found here are relevant to this mathematical model. These results will require testing against experimental data and until that time are conceptual in nature. In addition, a global sensitivity analysis of the dependence of network behaviours on model parameters is needed to assess the degree to which network behaviours depend upon parameter values. Finally, for networked models such as that presented here to be fully understood and reach their potential, it may be necessary to include their parallel or ‘event-driven’ implementations (unpublished [23]).

## Conclusion

The focus of this study has been on the adaptive response to unstable angina vs. recurrent MI of a networked model for autonomic cardiac regulation. While the network evolution is subject to global constraints, the network adaptations take place in a pathological setting that is outside prior experience. As such, the pathways followed by the system are described here as ‘free-floating’ implying that collective neuronal activities depend upon past events and changes in the network balance reflect experience accumulated from each myocardial ischemic episode. Details of the networked structure, the degree of neural diversity etc., all play a role in determining the system dynamics during pathology and the form of the network that emerges in the aftermath of pathology. The complexity of the dependence of neural network evolution upon network structure, neural diversity, and neural adaptability studied here for cardiac control is a current topic of wider interest within neuroscience involving questions of complexity and mechanisms for compensation in neural networks (e.g. [20–22] and [24]).

A benchmark tone that exists in the prelude to pathology among sympathetic efferent neurons, parasympathetic efferent neurons and central demand was used to compare the neural network internal balance before and in the aftermath of multiple pathologies. Stratified networks with relatively more peer-to-peer connections, compared with top-down networks that are more vertically integrated, exhibit a balance with relatively greater central demand inputs and significantly less sympathetic efferent neuronal tone. While higher neural diversity in stratified networks leads to somewhat greater resistance to sympatho-excitation than that seen in top-down networks of any neural diversity, there is no immunity to the combined effects of recurring pathology.

The cornerstone of investigative neurocardiology is the existence of local circuit neurons within the peripheral nervous system. The response of the network hierarchy as depicted herein, shows that the outcome of recurrent pathology strongly depends upon the degree to which local circuit neuronal populations are affected. In this study, a mathematical model was employed to consider the response of a network hierarchy to pathology. It highlights the degree of complexity that might be expected when recurrent pathology influences a neural network to seek out a new internal balance while continuing to satisfy ongoing needs for survival.

Many of the factors brought forth in the model simulations presented herein qualitatively substantiate preclinical and clinical observations. In the setting of ischemic heart disease, there are inherent and acquired factors that ultimately determine the potential for sudden cardiac death ([3] and [25]). While it is recognized that changes in the cardiac electrophysiological substrate are likewise important in arrhythmia generation ([26] and [27]), the concurrent effects on the cardiac nervous system and its autonomic efferent outflows reflect a critical aspect with morbitity and mortality consequences ([2, 3], and [28]). The critical nature of the local circuit neurons in modulation of the arrhythmogenic substrate has been demonstrated for both surgical and bioelectric therapeutic approaches ([28–30], and [31]). The fundamental contributions of inputs to neurons that transduce heart rate and blood flow demand alters both the structural and functional organization of all three levels within the control hierarchy are evident for arrhythmia management and the progression into heart failure ([3, 32, 33] and [34]). Derangements in such autonomic control are known to exacerbate the progression of the disease processes ([2, 8, 16], and [35]).

From the perspective of the mathematical model presented here, the progression of an internal network balance toward a new normal while under the influence of recurrent MI and/or unstable angina is a setting outside the prior experience of the network. As a result, the balance in the aftermath of pathology depends in a complex way upon internal neural network quantities that are difficult to measure concomitantly throughout this hierarchy *in situ*. Hence, it may be clinically useful to use a preventative approach where sympathetic tone is reduced and autonomic derangement is aggressively treated. While novel therapies to do so are beginning to be clinically applied ([2, 16], and [36]), mathematical models remain one means to understand why the adoption of a set of clinical practices may lead to improved outcomes in patients suffering from such disorders.
